# “*I feel like a guest on the other side*”: youth-identified challenges and solutions to transitioning from adolescent to adult HIV services in Western Kenya

**DOI:** 10.1080/09540121.2025.2591425

**Published:** 2026-02-17

**Authors:** Gladys Moraa Ontuga, Dorothy I. Mangale, Jayne Lewis-Kulzer, Zachary Arochi Kwena, Eliud Akama, Harriet Fridah Adhiambo, Sarah Iguna, Oketch Bertha Akinyi, Evelyne Nyandieka, Eunice Omondi, Felix Ochieng, Okoth Clinton Owino, Sophia Goldin, Bukusi Elizabeth Anne, Elvin Geng, Laura K. Beres, Lisa L. Abuogi

**Affiliations:** a Center for Microbiology Research, Kenya Medical Research Institute, Nairobi, Kenya; b School of Medicine, Washington University, St. Louis, MO, USA; c Department of Obstetrics, Gynecology, and Reproductive Sciences, University of California, San Francisco, CA, USA; d School of Nursing, University of Washington, Seattle, WA, USA; e Department of Pediatrics, University of Colorado, Denver, CO, USA; f Department of International Health, Johns Hopkins University Bloomberg School of Public Health, Baltimore, MD, USA

**Keywords:** good health and well-being, reduced inequalities, no poverty, quality education, sustainable cities and communities

## Abstract

Adolescents and young adults living with HIV (AYAH) face significant challenges when transitioning from adolescent to adult HIV services. Up to 50% of AYAH do not remain engaged in care after transition, which may result in virological failure. We sought to understand challenges to clinic transitions and youth-identified solutions to optimize health outcomes. We conducted three focus group discussions (FGDs) (*n* = 33) and a 4-day participatory, human-centered design (HCD) workshop as part of this qualitative sub study. AYAH recruited for FGDs between October and November 2020 were from health facilities in Kisumu County, Kenya. A subset of FGD participants and peer navigators were recruited for the HCD workshop. Participants were purposefully recruited to obtain diversity based on various demographic variables. We used a semi-structured guide covering HIV retention and clinic transition domains. The resultant audio files were transcribed, and transcripts analyzed inductively to identify emerging themes. During the HCD workshops, AYAH presented transition as a priority and engaged in journey mapping, story-telling and brainstorming to describe how transition happens, the challenges they experience and possible solutions. HCD analysis involved insight documentation, dialogue and rapid thematic analysis. AYAH who were between 14 and 24 years and were majority (56%) female, identified transition challenges including: limited knowledge about the transition process among providers; poor preparation for transition; complex organization and clinic flow in the adult HIV clinic; privacy and stigma concerns; challenging client–provider relationship dynamics; and concern for loss of relationships with peers. AYAH proposed approaches to improve the transition experience including early transition planning, peer transition support groups, peer-led transition, ongoing relevant health education during clinic visits and active support for acceptance of one’s HIV status. AYA highlighted individual, provider and facility-level challenges with care transition. The solutions identified by AYAH can guide interventions to enhance HIV treatment engagement, including adherence and retention, during the critical transition period.

**Trial registration:**

ClinicalTrials.gov identifier: NCT04432571.

## Introduction

Transition from adolescent-focused care to adult HIV care may be the most critical time to support retention and medication adherence among aging adolescents and young adults living with HIV (AYAH) (Njuguna et al., 2020a; Ritchwood et al., 2020). AYAH aged 15–24 years exhibit significantly higher rates of disengagement from care compared to younger adolescents aged 10–14 years and adults aged 25 years and older living with HIV (Wilson et al., 2019). Transition of care is a known contributor to poor HIV treatment outcomes among AYAH, yet there are few evidence-based transition models operating in Africa (Munyayi & Van Wyk, 2023; Ouedraogo et al., 2024; Petinger et al., 2025; Sánchez et al., 2021). To inform approaches that can improve HIV care engagement among transitioning AYAH, a deeper understanding of the obstacles and possible solutions from AYAH perspectives are needed.

Adolescent to adult care transition is defined as “the purposeful, planned movement of individuals with chronic physical and medical conditions from child/adolescent-centered to adult-oriented healthcare systems” and though a relatively new area of HIV care in Africa has recently been explored primarily using robust descriptive studies (Chew et al., 2025; Njuguna et al., 2022). Transition to adult HIV care often involves a change in clinic day, location and systems, as well as working with new clinicians and staff (Njuguna et al., 2022). Additionally, it almost universally involves AYAH assuming autonomy and taking on full responsibility for their HIV care with significant reductions in caregiver and clinician involvement (Njuguna et al., 2020b). Transition support for AYAH has been disproportionately explored in high-income settings and may include one or more components such as individualized care plans, (Chew et al., 2025) group transition programs, communication, psychological support (Jegede & Van Wyk, 2022), health and sexual education (Continisio et al., 2020) and mHealth approaches (Griffith et al., 2019; Zanoni et al., 2019). Examples of transition success from high-income countries, where transition to adult clinics is offered via personalized specialist care, demonstrate the importance of tailoring services to AYAH (Chew et al., 2025). Despite studies among AYAH in Africa demonstrating low preference for, and satisfaction with transition, these transition strategies and other evidence-based tools and procedural guidelines to support the transition process are not frequently utilized in African HIV programs (Chew et al., 2025; Jegede & Van Wyk, 2022; Njuguna et al., 2020b).

While there is recognition that the specific experiences of AYAH should be sought and prioritized to improve AYAH outcomes, and though there has been improvement in the engagement of a broad stakeholder base in designing and developing effective transition programs, insufficient input has been gathered from exclusively from AYAH in Africa regarding their preferences for, and experiences with transition to inform transition (Njuguna et al., 2020a; Tsondai et al., 2020). This study utilizes AYAH-driven data to better understand perceived and experienced barriers to transitioning and AYAH recommendations for optimizing transition procedures in western Kenya.

## Methods

### Study design

This study was carried out as part of formative research preceding the Adapt for Adolescents (A4A) trial (NCT04432571) in western Kenya. We conducted focus group discussions (FGD) and a four-day human-centered design (HCD) workshop with AYAH to adapt the planned study interventions and explore perspectives on adolescent HIV care to support retention and viral suppression. Transition experiences and challenges emerged as a key theme during this formative phase. Our study explored these experiences in detail and generated youth-identified ideas to improve the transition experience. Those findings are reported here while details on the trial and formative phase are published elsewhere (Abuogi et al., 2023; Akama et al., 2023; Eshun-Wilson et al., 2022).

### Study setting and population

This study was conducted in Kisumu County, Kenya, a high HIV-burden county with a prevalence of 16.3%, three times the national prevalence of 4.9% (Mahy et al., 2019). Study sites included three Ministry of Health facilities with free-standing, youth-focused adolescent HIV centers supported by clinical providers and peer supporters trained in HIV care for this population.

### Recruitment and sampling

The study recruited AYAH aged 14–24 years, who were aware of their HIV status, on ART, and had been the residents of Kisumu for at least six months. We purposefully selected 25 participants to include variation in factors we believed may influence perspectives on their HIV care experience (i.e., age, sex, marital status, and duration on ART) for focus group discussions. The HCD workshop included a subset of AYAH who participated in the FGDs and were willing to discuss their HIV status (*n* = 25) as well as peer navigators who were also AYAH, had experience delivering psychosocial support in youth clinics, and who were the trial intervention implementers (*n* = 8). Eligible participants were contacted by phone or in the clinic to obtain verbal informed consent, while parental/caregiver consent was secured for AYAH <18 years, along with their assent prior to participation.

### Data collection

Between 15th September 2020 and 1st October 2020, a total of 33 AYAH participated in either the FGDs (*N* = 25) or the HCD workshop (*n* = 22), with 14 participating in both. Additionally, eight peer leads participated in the HCD workshop for a total of 33 unique participants overall. All FGDs explored AYAH experiences in HIV care, ART adherence and care engagement challenges, as well as their experiences and preferences. FGDs were conducted using semi-structured guides developed by the research team and conducted by trained study staff in the preferred language of participants (Kiswahili, Dholuo, English). Two FGDs were conducted virtually via Zoom due to COVID-19 restrictions, while one was carried out in-person (when local COVID policies allowed). Participants in the virtual FGDs who lacked suitable devices to facilitate the session were provided with tablets, internet access at the study office, and individual rooms for privacy and social distancing. FGDs were audio-recorded and transcribed verbatim. The facilitator also took notes during the discussion. FGD participants received a one-time reimbursement of 500 Kenya shillings ($USD4).

The HCD workshop was held in-person between 12th and 15th October 2020, and was facilitated by three trained youth supported by trained adult research team members and an HCD expert (LB). AYAH engaged in a range of iterative, participatory activities (e.g., experience mapping, role plays, visioning) seeking to understand experiences with HIV care, preferences around potential trial interventions (Akama et al., 2023), key challenges and possible solutions. From those activities, transition emerged as a specific challenge and priority among AYAH. The facilitators introduced specific story-telling and brainstorming activities to understand the process of transitioning from youth to adult HIV clinics, challenges and potential solutions. The workshop data were captured in visualized activity outputs, workshop notes, participant-documented insight statements and daily debrief reflections. Participants received 500 Kenyan shillings daily as a reimbursement for their time.

### Data analysis

Transcripts were coded using both deductive and inductive coding. The deductive codes broadly included challenges facing AYAH in HIV care, recommendations for the trial interventions, transition challenges and suggested solutions. Findings on general HIV care challenges and intervention preferences are reported elsewhere (Akama et al., 2023). Analysis was led by an established qualitative researcher (ZK) with coding and categorization of the data into themes and subthemes completed by four others (GO, SI, BO, FA). Analysis involved iteratively refining the codebook, discuss coding experiences, and resolving coding challenges using categorization and constant comparison to identify dominant themes (Akama et al., 2023; Hennink & Kaiser, 2022). HCD data were analyzed by workshop facilitators using dialogue and affinity mapping to identify both thematic clusters and relationships between clusters. The results, highlighting transition challenges and responsive solutions, were summarized in a report and shared with AYAH peer navigator participants for feedback and confirmation in post-workshop meetings. Transition-specific data from both FGDs and the HCD workshop were further synthesized and summarized by the GO and LA into themes focused on barriers, facilitators and recommendations to improve transition.

## Results

Participants varied in age ranging from 14 to 24 years, with the majority (72.8%; *N* = 24), being between 18 and 24 years of age. About half of the participants were female (48.5%; *N* = 16), most (85%, *N* = 28) had completed four years of high school (i.e., secondary level) and 15% (*N* = 5) had transitioned to adult care at time of study participation (Table 1).

While only 15% of participants had already experienced transition, the majority of AYAH held negative perspectives on transition, with a limited number feeling positively or recounting favorable experiences. Themes regarding transition included: (i) limited knowledge and skills related to the transition process among providers, (ii) poor preparation for transition/low transition readiness among AYAH, (iii) complex organization/structure of the adult HIV care clinic, (iv) privacy and stigma concerns, (v) challenging client–provider relationship dynamics, and (vi) concern for loss of relationships with peers. AYAH generally demonstrated willingness to transition and shared their perspectives on approaches to improve the transition experience. They also reported elements they perceived would lead to successfully completing transition, such as (i) early transition planning, (ii) peer-led social support, (iii) integration of relevant health education in the adult HIV clinic visits, and (iv) supporting adolescents to accept their HIV status.

### Challenges associated with adolescent HIV care transition

#### Perceived limited knowledge, skills and preparation among providers regarding the transition process

AYAH felt that the service providers at the adolescent clinic lacked knowledge and skills on how to prepare adolescents for transition. This was attributed to poor training on youth-friendly practices and limited human resources in adult clinics. Youth perceived this poor provider preparation to be an important cause of poor AYAH preparedness to effectively navigate the complexities of the adult HIV care system with confidence.

#### Poor preparation for transition/low transition readiness among youth

For those who had transitioned to adult clinics, experiences often include feelings of being rushed through the process with insufficient preparation. Some also reported dissatisfaction with the transition process, feeling that they were given minimal consultation and time to understand or adapt to the changes. This lack of adequate preparation made the transition seem abrupt and uncomfortable, with major concerns about suddenly needing to independently manage aspects of their healthcare previously handled by caregivers such as scheduling visits, arranging transportation, financing care and handling medications. Those who had not yet transitioned also expressed concerns, expecting that their future transitions would lack timely preparation. Many AYAH felt psychologically and emotionally unprepared for the transition, fearing they would be overwhelmed, distressed, confused and anxious.

#### Complex and burdensome organization of adult HIV care

Perceived and experienced difficulties adjusting to the adult clinic’s structure were common. Participants noted that unlike the youth centers, adult clinics did not offer friendly or engaging environments, making them feel like “outsiders in adult clinics” instead of welcomed and comfortable. Several transitioned AYAH underscored that unlike in the adult clinic, in the adolescent clinic, a range of activities take place, including discussions on safe sex, disclosure, early pregnancy and family planning, which is a holistic approach to their overall well-being, empowering them to make informed decisions about their lives.

… but now going to the adult side again you find that you take drugs as you go, there is even no education there … they [transitioned adolescents] are not being taught but with us at the adolescent’s side we were being told to take a balanced diet, and or how to use condom …

(FGD Male AYAH Participant)

Additionally, transitioned AYAH found that adult HIV clinics were characterized by prolonged waiting times due to long queues. Individual patient-provider time was short owing to an emphasis on quick patient turnover to manage the higher client volume. In contrast, AYAH were used to shorter wait times and more individualized care in youth clinics.

You find that the services at the adult side are … slow than the services at the adolescents’ side … You can come in the morning, but you will leave at lunch time.

(FGD Male AYAH Participant)

#### Privacy and stigma concerns

AYAH perceived a lack of privacy in the adult clinic. Confidentiality worries arose from a shared waiting lounge and in general a physical layout of adult clinics that does not support privacy. This physical infrastructure, contrasted with the youth-friendly environments of specialized youth HIV clinics that provide dedicated, private spaces.

Part of their concern was due to age discrepancies with the older population, which the adolescents found difficult to navigate. Mingling with adults at the clinic, especially at the waiting area, was challenging, and many had concerns about potential perceived judgment from older adults due to the sensitive nature of HIV-related issues.

… When you go to adult clinic, you find adults seated there and they look at you and wonder how this young person has started engaging in sexual activities that led to them contacting HIV” those questionable looks scare me …

(Female HCD AYAH participant)

AYAH also revealed that they anticipated more stigma in the new clinics because of the healthcare providers’ communication style, which lacked approachability and understanding. They felt providers paid little attention to patients and seemed to be in a hurry to clear the queue which contributed to a perception of being less understood. AYAH felt that the absence of such youth-centric elements in adult clinics partly contributed to feelings of isolation and discomfort, particularly when addressing sensitive topics in group discussions.

#### Challenging client–provider relationship dynamics

AYAH emphasized the importance of provider–patient relationships to support remaining in care and maintaining treatment regimens. They described how the reduced time spent with providers in the adult clinic prevented the development of trusting relationships with providers. Of more concern to the AYAH were reports of unkind interactions with service providers in the adult clinic, unlike in the adolescent clinic where the providers are friendlier, which fueled distrust, and a growing disconnect between the youth and the new providers.

I have been removed from the youths and I am being taken to adult’s side of which I find it to be a challenge … Like the way I was interacting this side, and the way I am interacting this other side [adult side] is a bit different, I feel like a n outsider in the other side. I feel like an unwanted guest when I go there.

(HCD Male AYAH Participant)

#### Loss of relationships with peers/weakened peer support networks

AYAH anticipated and experienced a loss of friendships and supportive peer networks available in adolescent clinic. The abrupt end to relational ties with their peers and curtailed opportunities for connection through peer support groups caused experiences of isolation among AYAH who had already transitioned. AYAH recognized that the lack of support could lead to poor retention in care and non-adherence to medication. In addition, they felt that compared to the youth clinic, the adult clinic offered less support for building new bonds between clients.

… the WhatsApp group that we were with before we were transitioned, it should remain just to have your friends so that you can at least feel at home. But don’t leave the WhatsApp group because you have been transitioned to adult clinic just remain in there; you will get some comfort unlike when you leave the group you feel alone and have no one to support you …

(FGD Female AYAH Participant)

AYAH also discussed a few cases where clients’ family members were unsupportive of the care transition, wanting the AYAH to remain in the familiar youth clinic environment and comfort of being handled by familiar service providers.

### Facilitators of adolescent HIV care transition

#### Early transition planning

AYAH identified flexible and patient-centered transition planning in adolescent HIV clinics as an essential ingredient for successful transitioning. Many AYAH identified that early preparation of adolescents helped address any potential challenges while demystifying the process with structured timelines. They emphasized the importance of beginning transition preparation as early as when the adolescents are 10–15 years old to reduce uncertainty, boost confidence, and make the transition process much smoother and more manageable.

#### Peer-led social support

Youth clinic support groups were found to be instrumental in reducing feelings of isolation and encouraging AYAH to stay in care and adhere to their medication. These peer support networks, including informal assistance from older AYAH who had already transitioned, significantly eased the transition process by preserving supportive relationships. Experienced peers played a crucial role in helping newly transitioned AYAH navigate the adult clinic environment and adjust to their new circumstances, thereby enhancing the overall success of the transition.

Maybe you can just come to the support group and if you have any problem, you can share it up and you can get a solution. Or you can just talk of … if let us say that if today we are on the girl meetings, we can talk on how maybe to help a girl maybe during the case of rape what do you do? How girls can manage their lives, if you have an emergency; things like that.

(FGD Male AYAH Participant)

If you go to the adult clinic and find your peers who have been there for some time, they can help you get services quickly and show you around how things are done. Those adults don’t have time for helping someone.

(HCD Female AYAH participant)

#### Acceptance of HIV status

Several AYAH felt that the acceptance of HIV status not only empowers youth to better care for their health during transition but also fosters treatment adherence and enhance overall quality of life. Facilitators, peer supporters and the clinical team’s involvement play a pivotal role in helping youth accept their HIV status by creating a supportive environment, offering relatable experiences and empathy, professional expertise and medical guidance. Together, these roles contribute to the overall well-being and acceptance of youth living with HIV.

… Another thing that can motivate or make them go to the clinic most of the time is just someone to believe in himself or herself and accept the way he/she is …

(FGD Female AYAH Participant)

#### Suggested solutions for enhancing the transition experience

AYAH generated possible solution to some of the challenges they identified, with recommendations frequently highlighting facilitators that could be adapted to enhance the transition experience, making it more seamless and supportive. These included emphasizing the need for improved healthcare provider capacity building to address the unique needs of transitioning AYAH. Additionally, they stressed the importance of early transition preparation, advocating for comprehensive and timely education to ensure adolescents are well-informed and ready for the changes in adult care.

Integrating health education into clinic visits enhances patient–provider relationships and communication, allowing adolescents and young adults to share concerns and receive support. Several AYAH reported feeling that the health education available in adult clinics was inadequate.

Then at the adult clinic also, we are supposed to have something which can encourage someone to attend, like the doctors to be receptive … as in he/she should have those encouraging words which can make an adolescent be encouraged to attend the clinic there or have some sessions that are encouraging so someone can get motivated to go there.

(FGD Female AYAH Participant)

Notably, AYAH suggested a group cohort transition approach, emphasizing the benefits of transitioning AYAH in a supportive group setting. This approach fosters a sense of community and shared experiences, which can reduce isolation and disengagement. It also provides an opportunity for youth to ask questions and gain solidarity while navigating the complexities of adult care.

AYAH also highlighted the need for effective collaboration among transitioning youth, caregivers and the clinical team, both in adolescent and adult clinics. They underscored the involvement of all stakeholders, including clinic staff, parents/guardians and adolescents, as crucial for a smooth transition.

Finally, AYAH recommended developing detailed transition protocols, stressing the importance of clear guidelines and procedures to standardize and facilitate an effective transition process. Collectively, these recommendations aim to create an environment that not only supports a smoother transition but also prioritizes the overall well-being of AYAH as they move into adult healthcare.

**Table:** Summary of key themes related to the transition experience among adolescents and young adults with HIV (AYAH).

Category	Theme	Description
Barriers to Transition	Limited provider knowledge and skills	Providers lacked sufficient training, tools, and confidence to support structured transition
	Poor preparation/low readiness	AYAH reported feeling unprepared due to limited guidance, unclear expectations and abrupt timing
	Complex adult clinic organization	Adult HIV clinics were perceived as confusing, impersonal or overwhelming
	Privacy and stigma concerns	AYAH expressed fears about being recognized or judged, especially in adult clinic environments
	Client-provider relationship challenges	A lack of rapport, continuity and trust with adult providers negatively impacted comfort and care
	Concern over loss of peer relationships	Transition disrupted valuable peer connections established in adolescent-friendly clinics
Facilitators and AYAH Perspectives	Early transition planning	Initiating transition discussions and planning before the transfer age was seen as essential
	Peer-led social support	AYAH favored peer-led education and support groups to help normalize their experience
	Integrated health education	Continued education on HIV, treatment adherence, and life skills was recommended in adult care
	Support for HIV status acceptance	Psychosocial support to process and accept one's HIV status was viewed as critical to readiness
	Willingness to transition	Despite barriers, most AYAH expressed motivation to transition when supported appropriately

## Discussion

Findings from this qualitative study highlight key themes shaping the transition experiences of AYAH and youth-generated strategies proposed to improve this transition experience. Several transition challenges were reported by AYAH in this study including inadequate provider and patient preparation, difficulty adapting to different adult clinic culture and workflow, stigma, and loss of trusted provider and peer relationships. AYAH demonstrated a reluctance to transition citing discomfort with these challenges. However, they proposed potential solutions such as group transitions, earlier preparation and enhanced peer support, to facilitate a more friendly and supportive transition.

These findings are consistent with recent evidence from Sub Saharan Africa showing that the gap in implementation of effective transition approaches often stems from their poor contextual fit, limited provider training and lack of adolescent responsive services (Jegede & Van Wyk, 2022; Shimbre et al., 2024). Despite recommendations within the Kenyan National ART Guidelines, for example, encouraging a structured transition approach to transition, implementation at the clinic level remain inconsistent (Ministry of Health, National AIDS & STI Control Programme, 2022). Health system challenges including staff shortages and high turnover rates further exacerbate the problem, making the availability of providers trained transition difficult to maintain (Kinuthia et al., 2022). This mismatch between recommended practices and realword implementation may partially explain the lower rates of retention and viral suppression among transition age AYAH in many Africa settings.

AYAH in this study and others have identified the critical need for enhanced provider and patient preparation and well-defined clinic work flows to support the transition from adolescent to adult HIV care (Mbalinda et al., 2021). Many AYAH identified significant gaps in provider knowledge and training, which impact providers’ ability to facilitate a smooth transition. This aligns with the existing literature that underscores the importance of clinic policies and provider training to address transition needs (Jones et al., 2019; Njuguna et al., 2020a). Importantly, the absence of adequate preparation and support in adult clinics has been shown to negatively affect AYAH engagement in care (Njuguna et al., 2019; Ounchanum et al., 2024). AYAH in our study echoed these concerns describing poor communication, minimal guidance and lack of clarity about what to expect after transfer. Similar findings have been reported in Ethiopia and Kenya where undefined workflows and limited provider training identified as key barriers to effective transition (Shiferaw & Gebremedhin, 2020; Wanga et al., 2020). While structured transition processes offer opportunities enhance communication, skill building and continuity of care among AYAH and healthcare providers, successful implementation requires greater contextual adaptation and clear understanding on how to operationalize national guidelines in resource limited settings (Zanoni et al., 2021).

Additionally, AYAH in this study and others reported that transition often occur abruptly some based on age without adequate preparation or assessment of readiness, a practice that may contribute to disengagement (Njuguna et al., 2019). Several studies have identified the lack of use of transition readiness tools despite global recommendation supporting their use to build skills, promote autonomy and improve preparedness (Mbalinda et al., 2021; Njuguna et al., 2022; Zanoni et al., 2021). While transition readiness tools have been extensively employed in other settings for chronic illnesses to facilitate transition, their adoption remains limited in SSA (Parfeniuk et al., 2020; Varty & Popejoy, 2020).

AYAH in this study proposed thoughtful alternatives including structured preparation. This align with recent findings from a trial that demonstrated improved retention and viral suppression through a structured package combining readiness assessments, peer support and provider training (Njuguna et al., 2022). Our findings support the relevance of such intervention and underscore the value of youth driven, flexible approaches that go beyond aged based criteria. Tailored adolescent services have been encouraged to optimize care delivered for AYAH (Adams et al., 2022). Youth-friendly clinics that include supportive providers trained in adolescent care, dedicated clinics and waiting areas, and adolescent peer supporters have been advocated (Zanoni et al., 2021). While beneficial to address age-appropriate HIV treatment challenges, youth clinics ultimately require transition to adult HIV clinics (Zanoni et al., 2024). AYAH in our study, along with others in different studies, perceive the atmosphere in adult HIV clinics as “unfriendly”(-Zanoni et al., 2019). This perception is reinforced by longer waiting times and less patient-centered care. Interestingly, AYAH reported missing the age-appropriate health education relevant to their age group including sexual and reproductive health and age relevant counselling topics that were part of their adolescent clinic visits. AYAH strongly supported the continued integration of youth-friendly elements into adult services to ensure continuity and maintain engagement in care even after transition.

Psychosocial support has been recognized as key to achieving successful outcomes for AYAH (Ashaba et al., 2024). AYAH report that the loss of this support by disrupting existing provider and peer relationships serves as a significant reason for reluctance to transition. Similar to AYAH in other settings, our findings show that transitioning AYAH in western Kenya report receiving less social support in terms of peer support groups, counselling and adolescent-focused healthcare providers, in adult clinics compared to adolescent clinics (Ashaba et al., 2024; Ounchanum et al., 2024; Rungmaitree et al., 2022). Stigma and disclosure concerns further contributed to transition hesitancy (Mbalinda et al., 2021). AYAH emphasized that adolescent clinics provided not only clinic services but also a sense of belonging. The breakdown of support systems negatively affects both mental health and care engagement (Nduati et al., 2025). With some AYAH suggesting that having a dedicated clinic day in adult settings could help mitigate stigma they face from the adults who might judge them as being too young to be engaging in sexual activities leading to HIV infection. These findings reinforce the need for stigma sensitive clinic model that account for youth navigating stigma and complex emotional challenges.

Difficulties associated with adapting to the new adult clinic environment may introduce or exacerbate pre-existing challenges with staying engaged in HIV care (Rungmaitree et al., 2022). To address these barriers, AYAH offered creative and practical solutions, including early preparation to allow sufficient time for adolescents and young adults to adapt to the transition, which aligns with findings from South Africa and Kenya, where studies highlight the benefits of early planning for easing the shift to adult care (Hacking et al., 2019; Njuguna et al., 2022). AYAH also advocated for group transitions for youth, with mentorship from those who have already made the shift, which could strengthen peer support. This approach is backed by the literature showing that peer support and mentorship can enhance the transition process as it offers emotional reassurance while empowering AYAH through lived in experiences, helping reduce fear and normalize transition (Njuguna et al., 2022). AYAH’s focus on enhancing multidisciplinary coordination and fostering acceptance of HIV status aligns with best practices identified in systematic reviews, including research from southern Nigeria that emphasizes the need for structured and comprehensive care (Ayuk et al., 2020; Parfeniuk et al., 2020). Additionally, while structured programs are endorsed, researchers suggest incorporating flexibility to adapt them to individual needs, ensuring successful transitions (Øgård-Repål et al., 2023; Parfeniuk et al., 2020). Leveraging the lived experience and voices of AYAH to identify and refine solutions and inform the optimization of the transition experience is critical (Njuguna et al., 2020b). The alignment of youth-generated ideas with the existing research highlights the importance of involving young people in translating research into action. While researchers may struggle to identify priorities, youth can guide them and implementers in providing support that is both effective and valued by their peers, creating synergistic benefits of experience and efficacy.

### Strengths and limitations

This study contributes to the expanding literature on challenges and recommendations associated with the transition of AYAH to adult care, particularly in low- and middle-income settings. Notably that the AYAH-led nature of the HCD activities is a unique strength, allowing for transition to rise as a key priority that was not originally a focus for study investigators. This allows the parent study and future research to offer a more personalized and effective framework for supporting youth retention via successful transitions. In addition, including perspectives from AYAH who have undergone the transition process adds a crucial dimension of authenticity to the study, confirming pre-transition concerns raised by AYAH and enhancing the credibility and applicability of the findings. There were limitations to this study. Since transition was an emergent priority from the formative data, the research may not have allowed time and engagement on the topic to fully explore the breadth and depth of its meaning, challenges and solutions. Despite this, efforts were made to select participants representing various subpopulations of AYAH, aiming to capture a range of perspectives and enhance the study’s relevance in this population and in similar contexts.

## Conclusion

Transition from youth-focused to general adult HIV care is a critical moment for youth during a developmentally important stage of life. Improving transition is a priority among young people. Data support that improving transition is an opportunity to improve health outcomes. Enhancing provider and client preparation, supporting social bonds throughout changes in care delivery approaches, and improving care coordination are creative solutions to transition challenges proposed by youth that are also evidence-based. By adopting a systematic and structured approach to transition, and incorporating recommendations of AYAH themselves, it may be possible to create a more supportive and effective transition process for adolescents moving into adult HIV care.

## Figures and Tables

**Table 1.** Summary of demographic characteristics of participants (*N* = 33).

Participant characteristics	All (*N* = 33)
Sex	
Male	17 (51.5%)
Female	16 (48.5%)
Age (in years)	
14–17	9 (27.2%)
18–24	24 (72.8%)
Highest level of education attained	
University/college	2 (6%)
Secondary level	28 (85%)
Primary level	3 (9%)
Duration on ART (years)	
<10 years	11 (34%)
>10 years	21 (65%)
Transitioned to adult HIV clinic	5 (15%)
Married/living with partner	2 (6%)

## Data Availability

All anonymized data relevant to this study are readily available upon request from the corresponding authors and approval by the Kenya Medical Research Institute’s Scientific and Ethics Review Unit in line with the requirements of the Kenya Data Protection Act 2019.
